# Future of Rehabilitation Interventions for Rheumatic Patients in the Mediterranean Region

**DOI:** 10.31138/mjr.28.2.70

**Published:** 2017-06-27

**Authors:** Aysen Akinci, Gamze Kiliç

**Affiliations:** 1 Department of Physical Medicine and Rehabilitation, Hacettepe University, School of Medicine, Ankara, Turkey,; 2 Department of Physical Medicine and Rehabilitation, Afyon Kocatepe University, Afyonkarahisar Turkey

**Keywords:** rehabilitation, rheumatology, Mediterranean

## Abstract

Chronic rheumatic diseases can commonly lead to significant physical disability, reduced health-related quality of life and high economic burden for the societies. In the last decades and despite the availability of novel, effective medical treatment for specific rheumatic diseases, rehabilitation interventions do have a pivot role in improving function and psychological status in these conditions. Several systematic reviews and evidence based management recommendations suggest nonpharmaceutical rehabilitation management as an adjunct to medical therapy. The composition of rehabilitative interventions may extensively vary including therapeutic exercise, patient education, occupational therapy, orthoses, assistive devices, work rehabilitation and physical modalities. Exercise therapy is the main component of non-pharmacological treatment and strongly recommended in international guidelines but currently there is no consensus regarding intensity, frequency, or type of rehabilitation program for patients with rheumatic diseases. So, rehabilitation should be designed on a patient-centered basis in the context of multidisciplinary approach.

## INTRODUCTION

Rheumatic diseases are commonly leading to a significant impairment of physical function and health-related quality of life. Subsequently they are associated with a high economic burden on society with a total cost of $321.8 billion reported by the Medical Expenditure Panel Survey in patients with arthritis and other rheumatic conditions. ^1^ It is increasingly acknowledged that chronic rheumatic diseases can adversely affect work ability or working status. A recent study demonstrated that work disability is a major consequence of rheumatic diseases and occurs in about 49% of patients with rheumatoid arthritis (RA), 39% of patients with psoriatic arthritis (PsA) as well as 41% of patients with ankylosing spondylitis (AS). ^2,3^ The worldwide prevalence of rheumatic diseases ranges from 12–24%, ^4–6^ whilst the estimated prevalence of RA in many Mediterranean countries varies between 0.2–5%. ^7,8^ On the other hand, Behçet’s disease and Familial Mediterranean fever are more common in this area compared to Europe and America. ^9–11^

Rehabilitation work areas show significant differences within the Mediterranean countries. While neurological rehabilitation is primarily implemented in Israel, France and to some extent in Italy, rheumatology and orthopaedic rehabilitation are primarily implemented in Turkey and Spain. ^12^ In the literature, there is limited reported data available to assess rehabilitation interventions for rheumatic disease in the Mediterranean region. This might be attributed to the lack of rehabilitation services, economic difficulties, inadequate knowledge amongst rheumatologists for the beneficial effects of non-pharmacological treatment strategies as well as underestimation of the role of allied health professionals in the management of rheumatic diseases across Mediterranean countries.

Until now, a few studies have assessed the effect of climatic variables such as temperature, humidity and barometric pressure on rheumatic diseases including RA, AS and osteoarthritis (OA) in Mediterranean countries. A prospective study showed that low temperature and decreased atmosphere pressure increase the risk of joint pain in Spanish patients with RA and OA respectively. ^13^ More interestingly Staalesen Strumse et al ^14^ compared the effectiveness of the same rehabilitation program between two settings with different climatic conditions, namely Mediterranean and Norwegian settings in patients with AS. When the rehabilitation was performed in the former setting, the improvement in health status and spinal mobility outcomes were much better and sustained for longer. In addition, the proportion of ASAS20 and ASAS40 responders were higher in the Mediterranean group than in the Norwegian group. These results suggest that specific groups of patient rehabilitation may be more beneficial in a warm climate setting such as the Mediterranean region, providing the rationale for better designed studies in these countries.

Rehabilitation in rheumatic diseases is a difficult task that requires knowledge, expertise and patience, as most of these conditions are progressive and vary in clinical course with periods of remission and exacerbation. In rheumatic diseases, disease consequences such as pain, loss of range of motion, joint instability, muscle weakness, and fatigue accompanied by loss of function result in marked and severe impairments in daily activities and limit participation in society, family and working life. ^15,16^

Rheumatic diseases usually require lifelong treatment. Despite the availability of effective medical treatment, the adverse outcome of chronic rheumatic disease on patients’ lives is still substantial. So, effective and comprehensive multidisciplinary treatment strategies are crucial. In fact, rehabilitation programs combined with pharmacologic therapy can improve patient’s physical, psychological and social functioning and well-being, reducing in parallel the disability and health care costs associated with underlying conditions. ^17–20^ Moreover, last updated evidence based treatment recommendations including European League Against Rheumatism (EULAR) recommendation on early arthritis as well as American College of Rheumatology/Spondylitis Association of America/Spondyloarthritis Research and Treatment Network (ACR/SAA/SPARTAN) recommendation on AS and non-radiographic axial spondyloarthritis (nr-axSpA) indicate of non-pharmacological interventions as an adjunct. ^21,22^

The aim of rehabilitation intervention for rheumatic disease can be summarized in the following terms: reducing pain and discomfort, improving or maintaining current physical, psychological and social functions, compensating for loss of function, preventing or slowing disability, increasing independence and health-related quality of life, providing social integration and decreasing cardiovascular risk.

Rehabilitation program can be applied in many different ways according to the patient’s needs and availability of health care professionals in rheumatology clinics. Lately, a more holistic approach is preferred and the most commonly implemented rehabilitation strategy for people with rheumatic disease is comprehensive multidisciplinary team care (MTC) including psychologists, podiatrists, dieticians, social workers, nurses, vocational, physical and occupational therapist under the supervision of a rheumatologist and/or rehabilitation specialist. ^23,24^ A number of studies have demonstrated that MTC programs have a favorable effect on disability, disease activity, physical and psychosocial outcomes in patients with a wide range of rheumatic diseases. ^25–27^ In daily practice, such rehabilitation interventions can be provided in inpatient rehabilitation units, outpatient rehabilitation centers or long-term self-care management. Several studies have suggested that inpatient MTC delivered better health outcome in the short-term in patients with RA, AS, OA, as well as other non-inflammatory musculoskeletal diseases, including low back pain (LBP) and chronic widespread pain compared to outpatient MTC. ^28^ In contrast, others have demonstrated equivalent clinical effects between inpatient and daycare or outpatient team care programs. ^29,30^ Long-term effect of the treatment on disability or other health outcomes is yet unclear in patients with rheumatic disease. In a systematic review, it was suggested that either inpatient or outpatient MTC had limited effect on disability at 12 months, <12 months or 2 years of follow-up in patients with RA. ^23^ More recent reviews also indicated that after receiving inpatient MTC, mild as well as short-term improvements were observed in most clinical outcomes in patients with inflammatory rheumatic disease. ^24^ So, there is a strong need for studies to explore the long-term clinical effectiveness of MTC care on functional status, disability and other disease outcome in patients with rheumatic disease.

In the modern treatment era, the rehabilitation program should be designed as patient-centered using a multi-disciplinary approach. All patients should be assessed systematically for disease consequences, and individual goals of therapy should also be clearly defined before the rehabilitation intervention. To address these requirements, a structured approach towards rehabilitative management has been proposed by the World Health Organization International Classification of Functioning, Disability and Health (ICF). ^31^ This approach consists of two parts that have several components including body functions and structures, activities and participation and contextual factors (environmental and personal). Within the field of rheumatology, the ICF model is accepted to fit well in assessing the adverse long terms outcomes in several rheumatic diseases including RA, AS, OA and osteoporosis. ^32,33^ Thus, these structural approaches can contribute to the implementation of appropriate rehabilitation interventions which can eventually meet patient’s expectations and health professionals’ goals for optimal level of functioning.

The composition of rehabilitative interventions may vary, but, recently, in the rheumatology setting, therapeutic exercise, patient education, occupational therapy, orthoses, assistive devices, work rehabilitation and physical modalities are most often included in rehabilitation intervention. Although in daily practice these interventions can be applied as a single treatment modality, usually combination therapy is preferred.

*
-Therapeutic exercise* : In general, exercise is considered the cornerstone of the rehabilitative management of rheumatic disease. The primary aim of exercise programs is to maintain or increase joint range of motion, muscle strength, bone mineralization as well as aerobic capacity. Moreover, according to recent studies, exercise should be prescribed to improve cardiorespiratory fitness and reduce cardiovascular risk in patients with RA and other rheumatic diseases. ^34^ Although there is growing evidence on the effectiveness and safety of exercises in most rheumatic diseases, ^18,35^ there is still insufficient evidence to suggest the optimal type of exercise as well as the intensity, the duration, the number of repetitions or the frequency of the program. Such interventions could be performed through different types of exercise including land-based, water-based, home-based, individual and supervised exercises.
A Cochrane review article analyzed the effectiveness of physiotherapy interventions in the management of AS. Eleven studies were included in the data analysis. It was reported that, supervised exercise program was more effective than individualized home exercise programs and also group exercise combined with spa was superior compared to group exercise alone in patients with AS. ^36^ According to the ACR/SAA/SPARTAN treatment recommendation for AS and nr-axSpA, active physical therapy interventions such as supervised exercise is strongly recommended compared to passive physical therapy interventions including massage, ultrasound or heat. Taking into account the similar effectiveness of aquatic and land-based physical therapy with regard to disease outcomes as well as the fact that land-based therapy is more easily accessed than aquatic therapy, land-based physical therapy interventions is primarily recommended. ^21^
In RA, more recent reviews have indicated that dynamic exercises (aerobic and/or muscle strength training) exert a positive effect on function, quality of life, fatigue, psychological status and pain without any adverse influence on disease activity and/or radiologic damage. Currently, aerobic exercise combined with muscle strength training as routine practice should be recommend to patients with RA. ^35,37^ But, several factors including disease activity, joint damage, comorbidity or physical inactivity may represent barriers for patients to participate in exercise programs. Therefore, exercise prescription in RA should be tailored according to the patient’s functional ability, performance and cardiorespiratory fitness. ^38,39^
In patients with OA, exercise therapy including strengthening and aerobic exercise is the main component of non-pharmacological treatment and strongly recommended in most evidence-based international guidelines. ^18,40,41^ In the recent Cochrane review, high to moderate-quality evidence has reported moderately relief of knee pain and slightly improvement in physical function and quality of life in patient with knee OA. ^42^ Similarly, in patients with hip OA, the positive effects of the exercise program on hip pain and physical function has been supported by high quality evidence. But there is only low-quality evidence from three small studies supporting that exercise has small favorable influence on quality of life. ^43^ On the other hand, little evidence concluded benefit of exercise for patients with hand OA. ^44^
Taken all together, the increasing amount of evidence suggests the effectiveness and safety of exercise in most rheumatic diseases. However, there are difficulties in the interpretation of these studies. Currently no guidance regarding the more effective type of exercise intervention per disease phenotype is available. Moreover, exercise duration, frequency, type (e.g. home versus aquatic, individualized or group-based) vary widely between the reported studies. Therefore, optimal therapeutic exercise programs for patients with rheumatic diseases should be prescribed on an individualized basis, taking into account the level of pain, disease activity, functional limitations, baseline levels of cardiorespiratory fitness as well as the shared decision between the patient and doctor. ^38^ For example, some patients enjoy aquatic exercise but others do not; therefore, besides the disease related factors, the patient’s preference and expectations should be taken into consideration before prescribing the exercise program.
Tumor necrosis factor inhibitors (TNFi) can be accepted as a major breakthrough in the treatment of several rheumatic diseases. So far, a few studies have examined the effect of TNFi therapy combined with exercise program on disease outcome in patients with AS. One study by Yigit S et al. suggested that home exercises combined with TNFi had better outcome for a variety of parameters (e.g. functional capacity, joint mobility and quality of life) compared with TNFi therapy alone. ^17^ Another study showed that intensive group exercises combined with TNFi was more efficient in term of spine mobility and physical function in patients with AS. ^19^ These results suggest that intensive rehabilitation intervention is more efficient in patients previously stabilized with biological therapy.
- *Work rehabilitation* : A number of studies suggested that vocational rehabilitation programs can reduce job loss, absence and sick leave as well as increase work participation, productivity and job retention rates. ^45^ Therefore, early identification of work problems is of outmost importance to prevent work disability.
*
-Occupational Therapy* : A beneficial effect of occupational therapy programs including lifestyle changes, joint protection, environmental modification, energy conservation, usage of orthoses and adaptive device on physical function has been demonstrated in a number of studies in rheumatic disease. ^46^ A Cochrane review demonstrated strong evidence for positive effect of occupational therapy on functional ability in patients with RA. ^47^ Also, more recently occupational therapy, dynamic exercises and hydrotherapy have been recommended in addition to pharmacological treatments by update of the EULAR recommendations for the management of early arthritis. ^22^
- *Physical Modalities* : Despite extensive clinical experience with physical modalities including thermotherapy, therapeutic ultrasound, low-level laser therapy and TENS in patients with rheumatic disease, evidence-based data about their affectivity is scarce. ^20,48^ More high-quality studies are warranted to demonstrate the effect of physical modality intervention on functional ability and other disease outcomes in patients with rheumatic diseases.
- *Future rehabilitation intervention* : Recent studies have explored alternative tools to improve active participation of patients to rehabilitation intervention. A few small studies described good short-term efficacy of virtual reality intervention on pain reduction in patients with RA. ^49,50^ Also, another study reported that routine exercise was performed more effectively by using the virtual reality intervention in patients with AS. ^51^ A more recent review article concluded that smartphone applications may have positive effect on self-management application in patients with rheumatic disease. ^52^ Therefore, web-based and mobile health interventions may contribute to an increased adherence to rehabilitation intervention in patients with rheumatic disease.

In the last decade, the availability of new and effective medical treatment for specific rheumatic diseases is increased. However, rehabilitation interventions have a pivotal role in improving function and psychological status in this population. Several systematic reviews and also evidence-based management recommendations suggest rehabilitation management as an adjunct to medical therapy. ^21,22^ Moreover, rehabilitation programs have reported synergistic effects when used with medical treatments. ^17,19^ Hence, rehabilitation intervention is still considered as the mainstay of management of rheumatic disease, even in the era of new and effective pharmacotherapies. ^53^ In this respect, comprehensive and multidisciplinary rehabilitation interventions should become available and should be implemented as early as possible in the course of disease. Future research agenda should focus on the adequate description of early rehabilitation interventions to prevent deformities and disease progression, and their significance when used in combination with conventional and biologic disease-modifying drugs. In addition, particular care should be taken in the design of rheumatology rehabilitation facilities, the prioritization of patients and their needs, as well as the level and structure of service delivery organization in order to achieve better long-term outcomes for patients with systemic rheumatic diseases.
